# Peritoneal Hydatidosis

**DOI:** 10.1155/2010/714106

**Published:** 2010-08-08

**Authors:** Daniela Costamagna, Roberto Maiocchi, Annunziatino Zampogna, Amedeo Alonzo, Roberta Ambrosini, Alessandro Stecco, Francesca Mercalli

**Affiliations:** ^1^Second Division of General Surgery, Azienda Ospedaliero-Universitaria Maggiore della Carità, Corso Mazzini 18, 28100 Novara, Italy; ^2^Department of Radiology, Azienda Ospedaliero-Universitaria Maggiore della Carità, Corso Mazzini 18, 28100 Novara, Italy; ^3^Department of Pathology, Azienda Ospedaliero-Universitaria Maggiore della Carità, Corso Mazzini 18, 28100 Novara, Italy

## Abstract

Secondary peritoneal hydatidosis is caused by spontaneous or iatrogenic rupture of hepatic echinococcal cysts. We describe the case of a 65-year-old Tunisian male patient with previous history of liver hydatidosis who presented to our attention with subocclusive status. Imaging revealed a retrovesical hydatid cyst, adherent to the sigmoid colon. The treatment of choice was surgical removal of the cyst and the sigmoid colon. The patient is now being closely followed up.

## 1. Background

Hydatid disease or Echinococcosis is endemic in Mediterranean regions. The liver and lung are the most frequently involved organs. Peritoneal hydatidosis accounts for 13% of all abdominal hydatidosis. It is almost always secondary to seeding from spontaneous rupture of hepatic cyst or spillage of cyst fluid during previous surgery [1, 2]. It may occur in many different appearances: as isolated peritoneal lesions with the same characteristics of a hepatic cyst or as a massive peritoneal spread. Here we present the case of a Tunisian patient with pelvic localization of peritoneal hydatidosis and personal history of liver surgery for hepatic echinococcosis.

## 2. Case Presentation

**Figure 1:**
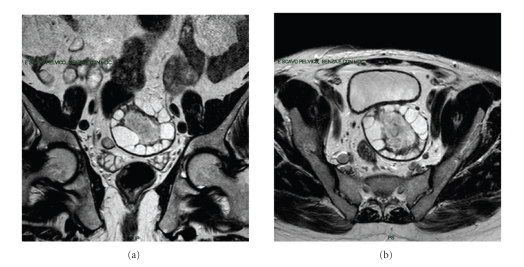
The MR images show the characteristic hydatid cyst in the retrovesical space, adherent to the sigmoid colon.

**Figure 2:**
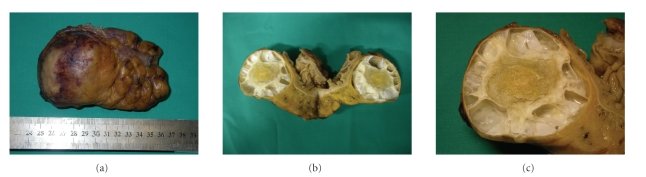
Surgical specimen.

**Figure 3:**
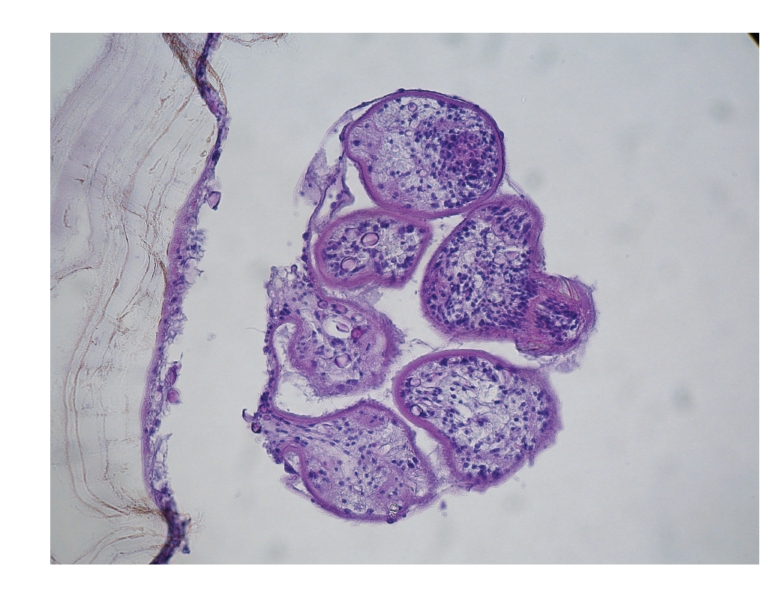
Microscopy findings.

In February 2010, a 65-year-old Tunisian male patient in good general conditions presented to our attention complaining from moderate abdominal pain and subocclusive status. In 1994, he underwent an operation for liver echinococcosis; the description of surgical procedure was not available. On the ultrasound examination and computed tomography, a diagnosis of pelvic hydatid cyst of 6.5 cm in diameter was made. In the preoperative planning, we decided to perform a magnetic resonance scan in order to study the relation of the cyst with contiguous organs (Figures 1(a) and 1(b)), and it was found to be posterior to the bladder and strictly adherent to the sigmoid colon. At laparotomy, the cyst was resected out “en bloc” with the sigmoid colon (Figures 2(a), 2(b), and 2(c)). Another small 2 × 1 cm cyst situated behind the bladder was removed. A lateroterminal mechanical anastomosis was performed. The histological examination confirmed the diagnosis (Figure 3). After the operation, the patient rapidly improved and was discharged on the 7th postoperative day. Albendazole was started 2 × 400 mg/day for 4 weeks. The patient is now under clinical and radiological followup. 

## 3. Discussion

Peritoneal hydatidosis is almost always secondary to a hepatic disease, in particularly related to previous surgery for liver cysts. Peritoneal involvement typically remains silent for years and is usually undetected unless cysts are large enough to cause symptoms. The differential diagnosis is usually not a problem. Ultrasonography and computed tomography are both excellent imaging modalities for the detection of hydatid cysts. The usefulness of magnetic resonance imaging lays in the precise definition of the anatomic relationship due to the excellent resolution for soft tissues [3]. The treatment of choice for localized peritoneal cyst is principally a careful and complete surgical excision [4, 5]. The treatment with albendazole may prevent the recurrence of cysts.
